# Sex dependent correlation of spleen atrophy and behavior deficits induced by binge treatment with ethanol in rodent models

**DOI:** 10.1515/nipt-2024-0016

**Published:** 2024-12-11

**Authors:** Jonathan Zhang, Muhammed Bishir, Wenfei Huang, Sulie L. Chang

**Affiliations:** Institute of NeuroImmune Pharmacology, 5971 Seton Hall University, South Orange, NJ, USA; Department of Biological Sciences, 5971 Seton Hall University, South Orange, NJ, USA

**Keywords:** ingenuity pathway analysis (IPA), gene expression omnibus (GEO) GSE49028, correlation analysis, differentially expressed genes pathway analysis, prefrontal cortex

## Abstract

**Objectives:**

During physical and psychosocial development, many adolescents engage in binge alcohol drinking. Ethanol (EtOH) is the key chemical in alcoholic beverages. EtOH intoxication impairs locomotor behaviors. We previously found that binge treatment with EtOH (BE) causes spleen atrophy, leading to immune dysregulation. With these premises, we hypothesized that BE-induced spleen atrophy is correlated with compromised locomotion and behaviors in adolescence.

**Methods:**

We exposed F344 rats to either 3 days of BE (mimicking college drinking) or water following pubertal onset. 24 h following the last BE, we assessed behaviors using ANY-Maze, focusing on locomotor activity, freezing, and thigmotaxis, before spleen collection. Correlation analysis and Linear Regression analysis quantified BE’s effects on behavior. In parallel, we used GEO2R to obtain differentially expressed genes (DEGs) from public dataset GSE49028 (B6129Sf2/J mice were given BE) and identified signaling pathways in the prefrontal cortex (PFC) involved in BE compromising locomotion and increasing anxiety.

**Results:**

BE significantly decreased spleen size. Interestingly, we found that BE exposure had a gender-dependent impact, affecting males more than females. Furthermore, functional analysis of the dataset identified several targets of interest including the downregulation of BDNF as a critical regulator of behavioral deficit following BE treatment.

**Conclusions:**

Using data-driven discovery and hypothesis-testing investigation to integrate these two studies, we provide an understanding of the underlying biological mechanism of BE-induced spleen atrophy-associated behavioral impairments through the genetic alterations in the PFC. Our findings will help develop a potent, powerful cocktail of reagents to treat behavioral impairment in those who binge drink.

## Background

Binge drinking is defined by the National Institute on Alcohol Abuse and Alcoholism (NIAAA) as consuming alcohol to achieve a blood alcohol concentration (BAC) greater than 0.08 % (0.08 g/dL). It affects 24 % of the US population [1], 2]. About 16 % of adolescents, 50 % of college students, and 44 % of high schoolers binge drink [2], 3]. Binge drinking is especially harmful in adolescence, a stage of physical and psychological development following the onset of puberty. Fewer drinks are required in youth (3 drinks for girls and 3–4 drinks for boys) to reach the same BAC as adults (4 drinks for women and 5 drinks for men) [4]. Consequently, it is easier for adolescents to develop health problems such as alcohol poisoning and alcohol-related blackouts and injuries.

Binge drinking is linked to poor cognition including learning, psychomotor speed, attention, executive function, and impulsivity [5]. Alcohol disrupts the immune system, impairing the body’s ability to combat infections by significantly weakening host defenses, leading to adverse immune-related illnesses like susceptibility to pneumonia [6]. The spleen, the largest immune organ, is involved in filtering blood, removing old or damaged erythrocytes and platelets, combating bacterial infection, activating the complement system, producing opsonizing molecules, storing monocytes and lymphocytes, activating B and T cells, and regulating immune homeostasis [7]. Our previous studies found differential spleen atrophy in adolescent F344 rats given binge treatment with ethanol (BE) [7].

Binge drinking modulates neuroinflammation via neuroimmune signaling connecting the peripheral and enteric nervous system to the central nervous system (CNS) [8], 9]. The bidirectional interactions between the nervous and immune systems involve the sympathetic nervous system, the hypothalamic-pituitary-adrenal (HPA) axis, and the splenic immune reservoir. The sympathetic nervous system is regulated by IL-1 and TNF-a cytokines produced by the splenic immune cells. In a feedback loop, the sympathetic nervous system modulates immune cells by releasing noradrenaline by the spleen [10]. In addition, the brain-spleen axis is mediated by the efferent vagus nerve which sends signals to the celiac ganglion and the spleen via the splenic nerve. The afferent vagus nerve receives inflammatory cytokines from the immune cells and antibodies which activate microglia and contribute to inflammation from the spleen and the efferent nerve response to the signals cholinergic anti-inflammatory signals from the brain via the efferent nerve [11].

The neuroimmune communication with the brain affects behavior and mood. Stress alters the immune responses via the neuroendocrine pathways. The microglia changes and monocyte priming leads to peripheral and central inflammation that contributes to the development of anxiety-like behaviors [12]. The development of anxiety is mediated through monocyte trafficking from the spleen to the brain in stress-sensitized mice, suggesting neuroinflammatory and neuroimmune mediated by the brain-spleen axis [13]. Lisboa et al. administered endocannabinoid receptor agonists to C57BL/6 mice for six days, resulting in increased spleen size, decreased anxiety-like behavior, and decreased circulating monocytes and neuroinflammation markers in the brain [14]. Excessive intermittent exposure to alcohol elevates forebrain glucocorticoid signaling, which damages the PFC, a critical area for executive functions as well as anxiety and motor control [15], [16], [17]. The spleen is important in regulating neuroimmune and neuroinflammatory mechanisms contributing to behavioral changes.

We have previously shown that BE exposure in male adolescent F344 rats causes spleen atrophy and induces a stress response mediated by the HPA axis and hippocampus [7]. With these solid premises, we have hypothesized that spleen atrophy contributes to behavioral deficits. We have integrated (1) an *in-vivo* study using F344 rats given 3-day BE treatment and (2) an in-silico study using differentially expressed genes (DEGs) obtained from GSE49028 dataset from young adult mice given single BE treatment. We studied sex-dependent differential spleen weight change in F344 rats and correlated with behavioral deficits including anxiety and locomotor impairment. These findings suggest that BE-induced spleen atrophy in adolescents may lead to anxiety and locomotor deficits. We analyzed the PFC of mice given BE treatment through functional and pathway bioinformatic analysis of the DEGs to study the molecular mechanisms underlying BE-induced behavioral changes. The pathways and regulators being identified reveal potential therapeutic drug targets for treating BE-induced spleen atrophy-associated behavioral deficits in adolescents exposed to binge drinking.

## Methods and materials

### Animals

48 F344 rats (24 male, 24 female) were purchased from Envigo (Indianapolis, IN). Three rats were housed per ventilated plastic cages (Animal Care Systems Inc., Centennial, CO). Rats were in a temperature- and humidity-controlled environment with a 12-h light/dark cycle and given *ad libitum* access to a standard rat diet and water. The animals were monitored, and their body weight was recorded daily. The experimental protocol was approved by the Institutional Animal Care and Use Committee (IACUC) at Seton Hall University.

As reported by Farris et al. [18], 12-week-old male B6129SF2/J mice (n=18) were from Jackson Laboratories. Mice (4–5) were housed per cage. Mice had free access to water and standard rodent chow on a 12 h light/dark cycle. Mice were acclimated to the facility for 1 week before treatment. Animals were treated and handled according to IACUC protocols approved by Virginia Commonwealth University and the National Institute for Health.

### EtOH administration and tissue collection

The F344 rats arrived on postnatal days (PND) 21. Rats were conditioned to intragavage (i.g.) administration one week before expected pubertal onset (male PND 45–66 and female PND 35–62). Followed by either 3 days of BE at 4.8 g 52 % w/v EtOH (9.23 mL/kg) (male n=15 and female n=15) or the equivalent volume of water (male n=9 and female n=9) starting on the day of pubertal onset. The rats were sacrificed 20 h after the last treatment followed by necropsy. Spleens of each rat (male water n=9, male EtOH n=9) were collected, weighed, and calculated as relative spleen weight (RSW) determined as spleen weight (mg)/body weight (g). The EtOH-treated rats were subdivided into three EtOH groups for each sex sorted into low RSW change (FemaleE1 and MaleE1), moderate RSW change (FemaleE2 and MaleE2), and high RSW change (FemaleE3 and MaleE3) for subsequent analysis.

As reported by Farris et al., B6129SF2/J mice were treated with intraperitoneal saline injections (n=9) or 3 g/kg (20 % v/v, binge level) of EtOH (n=9). After scarification at 4 h after treatment by cervical dislocation and decapitation, the prefrontal cortex (PFC) was collected for RNA sequencing [18].

### Locomotor open field assessment

F344 rat’s locomotor behavior was measured in the open field apparatus (Stoelting Co, Wood Dale, IL), to track animal movements using ANY-maze video software (Stoelting Co, Wood Dale, IL). 20 h following the third BE treatment, F344 rats were placed in the open field apparatus for 10 min. The open field apparatus was divided into 16 equal quadrants (4 × 4), where the outer 12 quadrants were labeled as the periphery zone and the inner 4 quadrants were labeled as the center zone. This assessment measures distance traveled, mean speed, number of freezing episodes, freezing time, time mobile, number of mobile episodes, center zone entries, center distance traveled, periphery zone entries, and periphery zone distance. Mean speed is a measurement of locomotor activity. Freezing is an instinctive fear response measured by freezing and mobility. Thigmotaxis, also known as wall hugging, is associated with anxiety and less exploratory behavior measured by zone entries and distance.

### Statistical analysis

Statistical analysis was performed using IBM SPSS software Version 29 (IBM Corp, Armonk, NY). Pearson correlation analysis was conducted between the RSW and the open field behavioral assessments (total distance traveled, mean speed, number of freezing episodes, time freezing, mean freezing score, time mobile, number of mobile episodes, number of center zone entries, center distance traveled, number of periphery zone entries, and periphery distance traveled). *t*-test and Linear regression analysis were conducted on water- and EtOH-treated F344 male and female rat RSW versus various behavioral measurements (mean speed, freezing score, and center zone distance). For all analyses, a p-value of less than 0.05 was considered significant.

### Transcriptome profile of acute EtOH administration of B6129SF2/J mice

The microarray data of B6129SF2/J mice treated with BE or saline as reported by Farris et al. were collected from the National Center for Biotechnology Information’s Gene Expression Omnibus (GEO) under accession GSE49028 [18]. The differentially expressed genes (DEGs) from the EtOH-treated mice compared to the control saline-treated mice were obtained from GSE49028 using GEO2R. GEO2R is part of NCBI’s public tool that compares two or more groups of samples to identify genes that are differentially expressed across experimental conditions.

### Ingenuity pathway analysis (IPA) software

The IPA Analysis Match CL license was purchased from QIAGEN for using all features and tools of the IPA 22.0 software. Data analysis for this study was conducted between 1 April 2023 and 10 August 2024 using IPA and QKB (QIAGEN Inc., Hilden, North Rhine-Westphalia, Germany https://www.qiagenbioinformatics.com/products). QKB is a database of over 12 million literature findings from about 3,600 peer-reviewed journals curated by over 2,000 QIAGEN scientists. These literature findings are experimentally demonstrated. Furthermore, information from third-party databases such as BioGRID, ClinGen, and PubChem are manually reviewed and incorporated into QKB. Experimental data are taken from the literature articles, where the interactions are between molecule and molecule, molecule and cell and cellular process, molecule and disease, molecule and tissue, and molecule and phenotype. The molecule refers to any gene, RNA, protein, or chemical. The database covers mammalian information from humans, mice, and rats.

### Differentially expressed genes pathway analysis

IPA Core Analysis was conducted on the DEGs obtained from the PFC of GSE49028. The Core Analysis was conducted with the biological filter for the Nervous System. The Core Analysis tool identifies enriched relationships, mechanisms, and pathways. These canonical pathways are well-characterized metabolic and signaling pathways that are curated and hand-drawn based on pathways from peer-reviewed journal articles, review articles, textbooks, and HumanCyc for metabolic pathways. The pathway analysis determines the significance of the canonical pathway through a p-value that is calculated from the right-tailed Fischer’s exact test. This p-value of overlap represents the probability of association of molecules from the dataset with the canonical pathway by random chance alone. The pathways related to behavioral deficits were selected for analysis to identify the downstream effects of the differentially expressed genes within the canonical pathways. Using IPA’s overlay tool, the gene regulation of the DEGs was visualized on the signaling pathway with red representing upregulation and green representing downregulation where the intensity of the color represents the magnitude of the DEGs’ fold change.

### Differentially expressed genes functional analysis

IPA’s Core Analysis allows for functional analysis to associate biological functions and diseases within the dataset by identifying Diseases & Functions that are found to be involved in the dataset. The z-score was obtained from both the pathway analysis and functional analysis to represent the strength of activation (positive z-score) or inhibition (negative z-score) of the canonical pathway or Diseases & Functions. Z-scores ≥2 indicate that the disease or function is statistically significantly increased. Z-scores ≤−2 indicate that the disease or function is statistically significantly decreased. A p-value of less than 0.05 was used to filter out the Diseases & Functions. Eight of the 834 Diseases and Functions significantly identified in the GSE49028 were involved in the role of anxiety (Anxiety, Exploratory Behavior, Emotional behavior, and Cognitive impairment) and locomotor deficit (Motor dysfunction, Myelination of nerves, and Motor learning). Disease and Functions involved in the role of anxiety and locomotor deficit were selected. All the selected Diseases and Functions were significantly (p-value <0.05) involved in the PFC of BE B6129Sf2/J mice.

## Results

### Binge EtOH-induced differentially RSW in F344 rats

**Figure 1:**
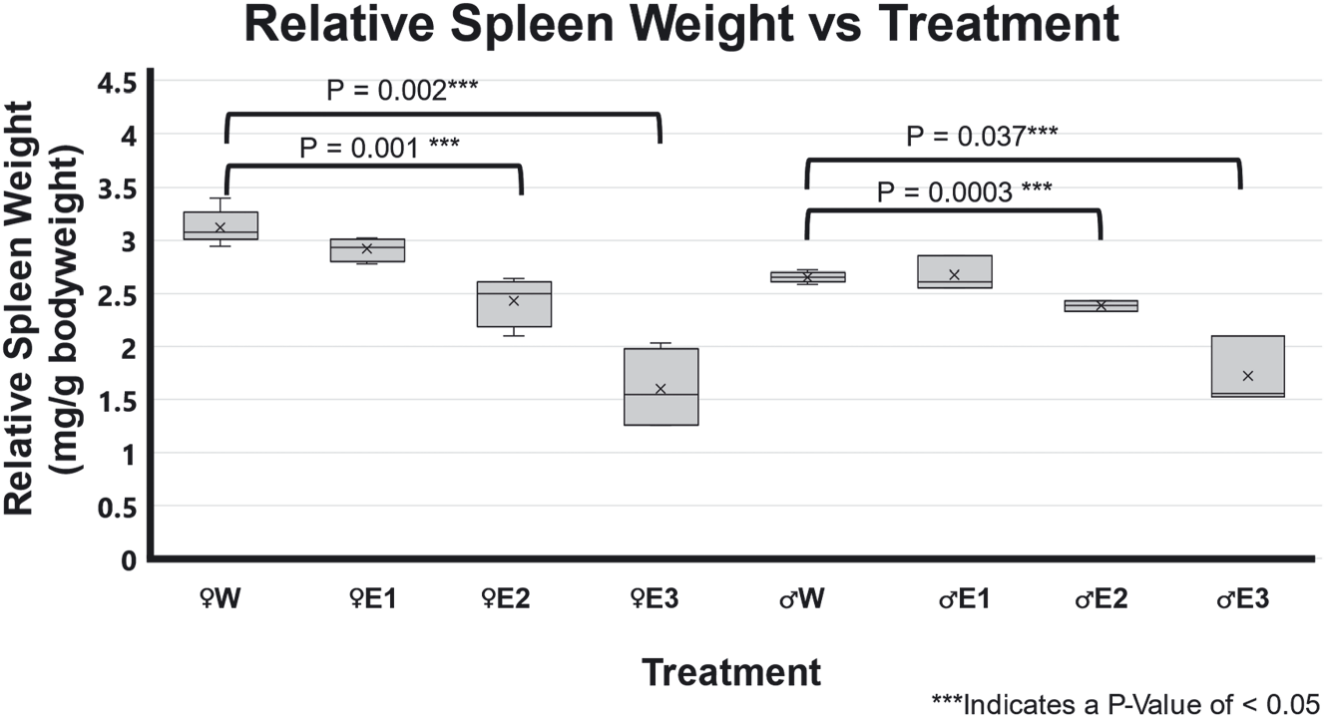
Impact of 3-day binge EtOH on relative spleen weight. Three days of binge EtOH treatment led to sex-dependent differential spleen weights in both male and female F344 rats. In females (♀), EtOH-treated groups had significantly lower relative spleen weights E2 (p=0.001) and E3 (p=0.002) compared to water-treated groups. Similarly, in males (♂), relative spleen weights in E2 (p=0.0003) and E3 (p=0.037) were significantly lower compared to water-treated controls.

The BE-treated rats from both male and female groups had significantly smaller spleen sizes than the water-treated animals (Figure 1). The EtOH-treated groups were subdivided into low RSW change (FemaleE1 n=4 and MaleE1 n=4), moderate RSW change (FemaleE2 n=4 and MaleE2 n=3), and high RSW change (FemaleE3 n=4 and MaleE3 n=3). The moderate RSW change (E2) in both male and female groups that were given BE treatment was significantly lower than the water-treated male (p=0.003) and female (p=0.001) groups. The high RSW change (E3) in both male and female groups that were given BE treatment was significantly lower than the water-treated male (p=0.0037) and female (p=0.002) groups.

### Sex-dependent correlation differences between spleen weight and behavioral measurements

Table 1 shows the Pearson Correlation Analysis compared among the female EtOH, female water, male EtOH, and male water groups. The RSW of EtOH-treated groups was significantly correlated with behavioral measurements in a sex-dependent manner stronger in males than females, while there were no significant correlations in the water-treated groups.

**Table 1:** Correlation analysis between relative spleen weight and locomotor activity measurements.^a^

	Treatment		Total distance traveled, m	Mean speed, m/s	# of freezing episodes	Time freezing, s	Mean freezing score	Time mobile, s	# of mobile episodes	# of center entries	Center distance traveled, m	# of periphery entries	Periphery distance traveled, m
Relative	Female	Pearson correlation	0.540^b^	0.538^b^	0.037	−0.481	0.491	0.468	0.341	0.222	0.289	0.237	0.536^b^
spleen	ethanol (n=14)	Sig. (2-tailed)	0.046	0.047	0.901	0.081	0.075	0.092	0.232	0.445	0.317	0.415	0.048
weight,	Female	Pearson correlation	−0.060	−0.063	0.096	−0.111	0.112	−0.060	0.025	0.134	−0.040	0.123	−0.059
mg/g	water (n=9)	Sig. (2-tailed)	0.878	0.871	0.806	0.776	0.774	0.879	0.948	0.731	0.919	0.752	0.881
body	Malee	Pearson correlation	0.745^b^	0.742^b^	−0.485	−0.725^b^	0.734^b^	0.639	0.765^b^	0.764^b^	0.609	0.741^b^	0.756^b^
weight	thanol (n=9)	Sig. (2-tailed)	0.021	0.022	0.186	0.027	0.024	0.064	0.016	0.017	0.082	0.022	0.018
	Malew	Pearson correlation	−0.318	−0.317	0.192	0.399	−0.302	−0.284	−0.128	−0.334	−0.577	−0.294	−0.187
	ater (n=9)	Sig. (2-tailed)	0.405	0.406	0.620	0.287	0.429	0.458	0.743	0.380	0.104	0.442	0.630

^a^The Pearson correlation coefficient (r) measures the linear relationship between two variables from −1 to 1 representing a strong positive or negative relationship respectively. A Pearson correlation near zero represents a nonlinear relationship. 0.7≤r<1 and −1<r≤−0.7 represents strong correlations, 0.5≤r<0.7 and −0.7<r≤−0.5 represents moderate correlations, and 0.3≤r<0.5 and −0.5<r≤−0.3 represents weak correlations. ^b^Correlation is significant at the 0.05 level (2-tailed). Correlation is significant at the 0.01 level (2-tailed).

The RSW of male EtOH group compared to female EtOH-treated group showed a stronger significant positive correlation with total distance traveled (in males Pearson correlation=0.745, p=0.021 and in females Pearson correlation=0.540, p=0.046) suggesting a smaller RSW was correlated with less total distance traveled.

The RSW of male compared to female EtOH-treated group had a stronger significant positive correlation with mean speed (in males Pearson correlation=0.742, p=0.022 and in females Pearson correlation=0.538, p=0.047).

The RSW was significantly correlated with time freezing (Pearson correlation=−0.725, p=0.027) and mean freezing score (Pearson correlation=0.734, p=0.024) in the male EtOH-treated group, but not significantly correlated in the female EtOH-treated group. The RSW was significantly correlated with the number of mobile episodes (Pearson correlation=0.765, p=0.0216) in the male EtOH-treated group, but not significantly correlated in the female EtOH-treated group.

The RSW was significantly correlated with the number of center entries (Pearson correlation=0.764, p=0.017), and the number of periphery entries (Pearson correlation=0.741, p=0.022) in the male EtOH-treated group, but not significantly correlated in female EtOH-treated group. The RSW had a stronger significant correlation with periphery distance traveled in males (Pearson correlation=0.756, p=0.018) than in females (Pearson correlation=0.536, p=0.048).

### Locomotor activity was correlated with RSW in binge EtOH-treated F344 rats

**Figure 2:**
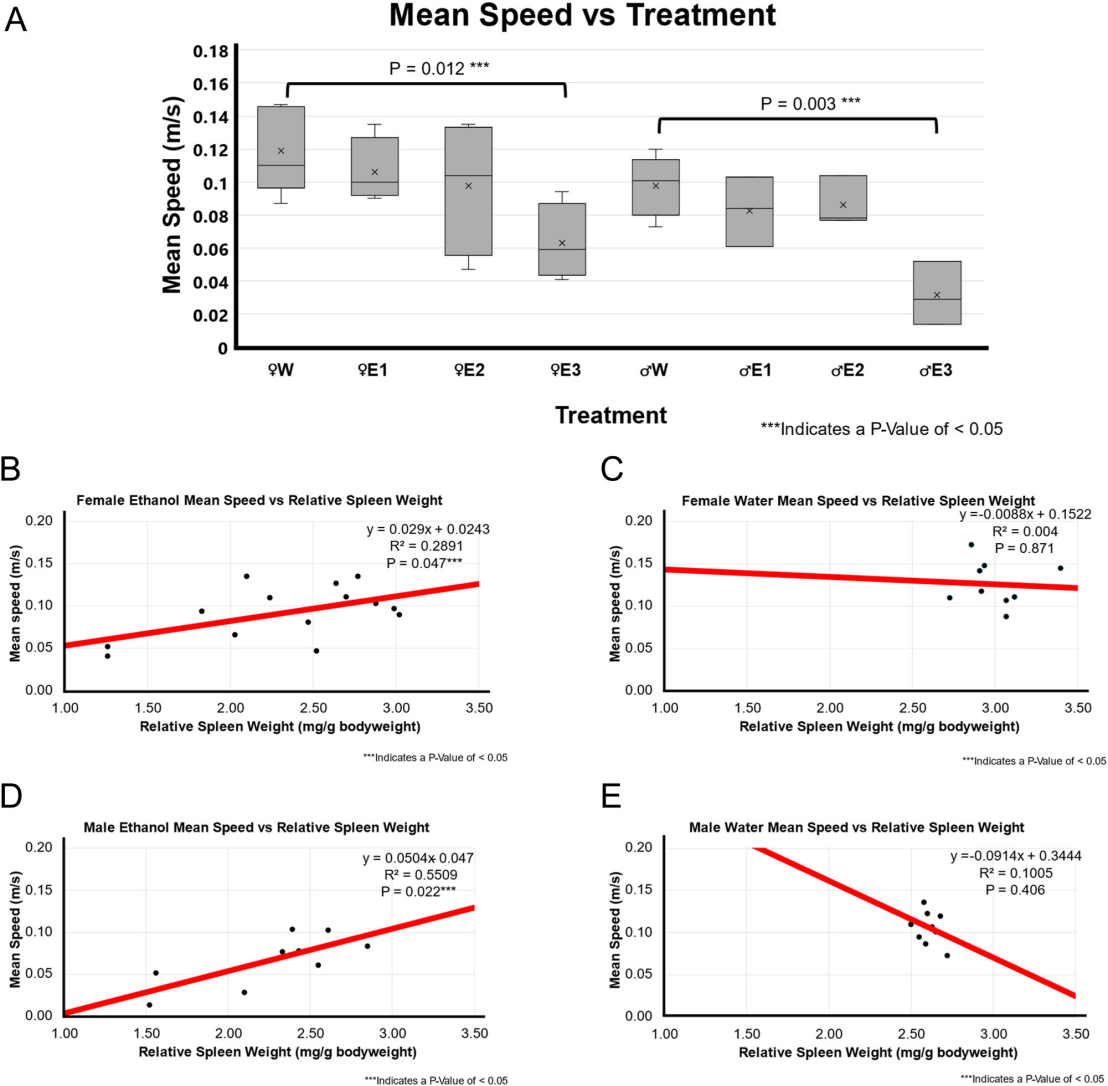
Mean speed and correlation with relative spleen weight following 3-day binge EtOH exposure. (A) Mean speed by treatment group. The relative spleen weight between the treatment groups was compared using a *t*-test between water (n=5 for male and n=5 for female) and EtOH-treated rats for both males (n=10) and females (n=12). In females, animals with smallest spleen weight following EtOH exposure, E3 showed significantly lower mean speed than the female water-treated group (p=0.012). Similarly, the male E3 EtOH-treated group showed significantly lower mean speed than the male water-treated group (p-value of 0.003). The spleen groups E2 and E3 also showed a decreasing trend in mean speed of EtOH-treated male and female F344 rats in OFT. (B) A positive weak correlation r^2 of 0.289 and p-value of 0.047 between relative spleen weight and mean speed in EtOH-treated female F344 rats. (C) A weak or no correlation (r^2 of 0.004; p=0.871) between relative spleen weight and mean speed was observed in water-treated female F344 rats. (D) Correlation between relative spleen weight and mean speed obtained by ANYmaze open field assessment in EtOH-treated males (n=9). They were significantly positive correlation (r^2 of 0.5509; p=0.022) smaller spleen weight and mean speed in EtOH-treated male F344 rats. (E) No to very weak correlation (r^2 of 0.1005; p-value of 0.406) was found between relative spleen weight and mean speed in water-treated male F344 rats.

BE-treated rats with a high RSW change had significantly less locomotor activity compared to water-treated rats in both females (p-value=0.039) and males (p-value of 0.020) as shown in Figure 2A.

There was a very weak neutral association in water-treated groups (female p-value of 0.871 and r^2 of 0.004, male p-value of 0.406 and r^2 of 0.1005) as shown in Figure 2C and E. However, there was a significant positive weak to moderate association between EtOH groups (female p-value of 0.047 and r^2 of 0.2891, male p-value of 0.022 and r^2 of 0.5509) as depicted in Figure 2B and D. This suggested that in EtOH groups, a smaller spleen weight was correlated with less mean speed.

### Fear response was correlated with RSW in binge EtOH-treated F344 rats

**Figure 3:**
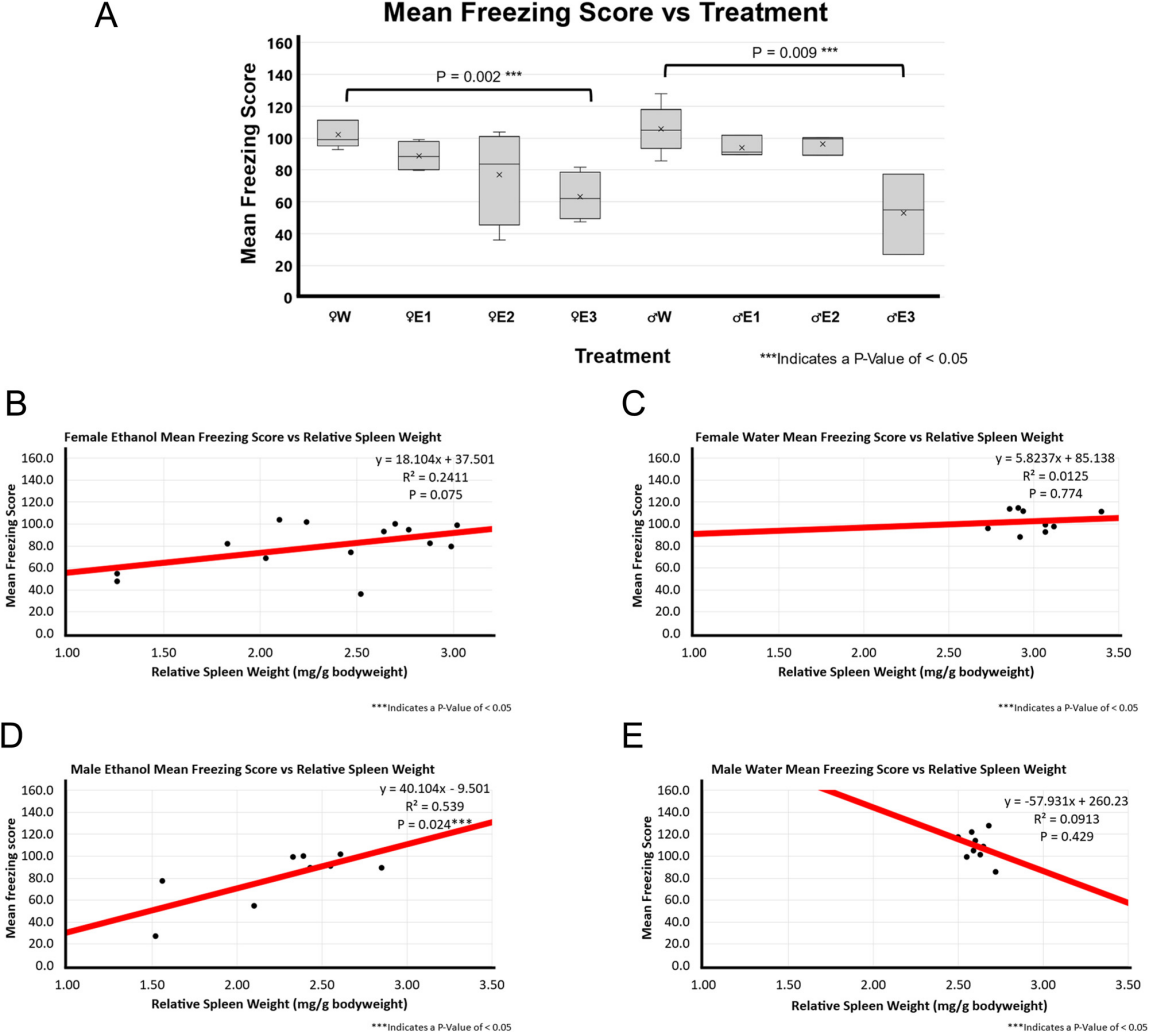
Mean freezing score and correlation with relative spleen weight following 3-day binge EtOH exposure. (A) Mean speed by treatment group. The relative spleen weight between the treatment groups was compared using a *t*-test between water (n=5 for male and n=5 for female) and EtOH-treated rats for both males (n=10) and females (n=12). In females, animals with smallest spleen weight following EtOH exposure, E3 showed significantly lower mean freezing score than the female water-treated group (p=0.002). Similarly, the male E3 EtOH-treated group showed significantly lower mean freezing score than the male water-treated group (p=0.009). The spleen groups E2 and E3 also showed a decreasing trend in mean speed of EtOH- treated male and female F344 rats in OFT. (B) A positive weak correlation (r^2 of 0.2411; p=0.075; n=14) was found between relative spleen weight and mean speed in EtOH-treated female F344 rats. (C) A weak to no correlation (r^2 of 0.0125; p=0.74; n=9) between relative spleen weight and mean freezing score was observed in water-treated female F344 rats. (D) Positive moderate correlation (r^2 of 0.539; p=0.024; n=9) was found between smaller spleen weight and mean freezing score in EtOH-treated male F344 rats. (E) No to very weak correlation (r^2 of 0.0913; p-value of 0.429; n=9) was found between relative spleen weight and mean speed in water-treated male F344 rats.

The mean freezing score was calculated based on the time spent freezing and the number of freezing episodes, where a lower freezing score indicates a greater freezing response. BE rats with high RSW change had a significantly lower mean freezing score than water-treated rats in both female (p-value of 0.002) and male (p-value of 0.009) groups as shown in Figure 3A.

Figure 3B–E depict the correlation analysis between RSW and mean freezing score in the different treatment groups. There was a very weak or weak association in both water and BE groups (female EtOH p-value of 0.075 and r^2 of 0.241, female water p-value of 0.774 and r^2 of 0.013, and male water p-value of 0.429 and r^2 of 0.091) except male BE group. There was a significant moderate positive association in the BE male rats (p-value of 0.024 and r^2 of 0.539).

**Figure 4:**
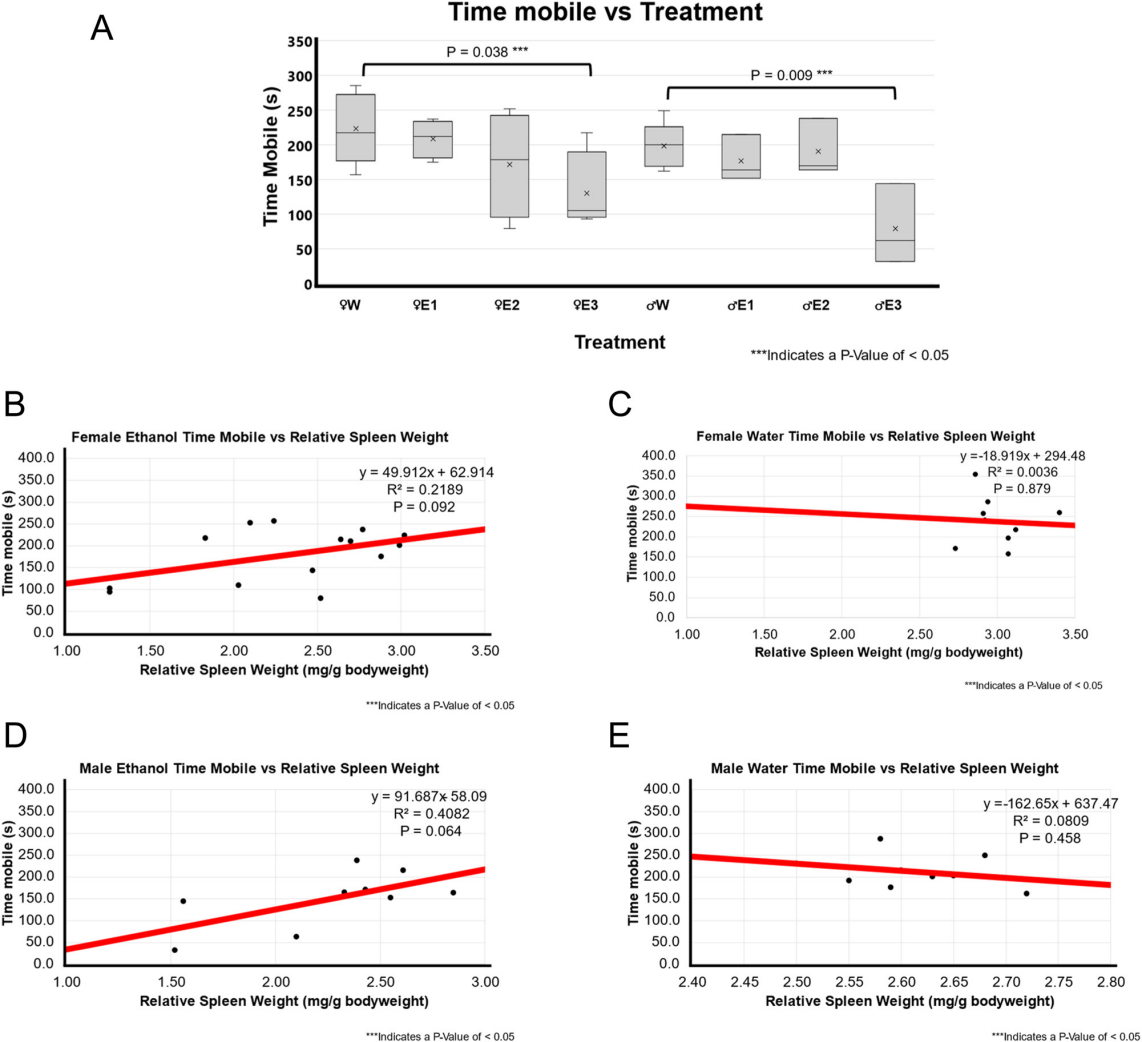
Locomotor activity-time mobile and correlation with relative spleen weight following 3-day binge EtOH exposure. (A) The relative spleen weight between the treatment groups was compared using a *t*-test between water (n=5 for male and n=5 for female) and EtOH-treated rats for both males (n=10) and females (n=12). In females, animals with smallest spleen weight following EtOH exposure, E3 showed significantly less time mobile compared to female water-treated group (p=0.038). In males, smallest spleen group E3 group showed significantly lower time mobile compared male F344 rats treated with water (p=0.009). (B) A positive weak correlation (r^2 of 0.2198; p=0.092; n=14) was found between relative spleen weight and time mobile in EtOH-treated female F344 rats. (C) A weak to no correlation (r^2 of 0.0036; p=0.879; n=9) between relative spleen weight and time mobile score was observed in water-treated female F344 rats. (D) Positive moderate correlation (r^2 of 0.4082; p=0.064; n=9) was found between smaller spleen weight and mean freezing score in EtOH-treated male F344 rats. (E) No to very weak correlation (r^2 of 0.0809; p-value of 0.458; n=9) was found between relative spleen weight and mean speed in water-treated male F344 rats.

Figure 4 compares the mobility of different treatment groups as an indication of fear through the amount of time spent mobile. Both male and female EtOH-treated rats spent less time mobile than water-treated rats, this was especially significant in high RSW change (female p-value of 0.038 and male p-value of 0.009).

There was a very weak or weak neutral association in both water and BE groups for (female EtOH p-value of 0.092 and r^2 of 0.219, female water p-value of 0.879 and r^2 of 0.004, and male water p-value of 0.458 and r^2 of 0.081) except male BE group. There is a moderate positive association in the BE male rats (p-value of 0.064 and r^2 of 0.408).

### Anxiety response was correlated with RSW in binge EtOH-treated F344 rat

**Figure 5:**
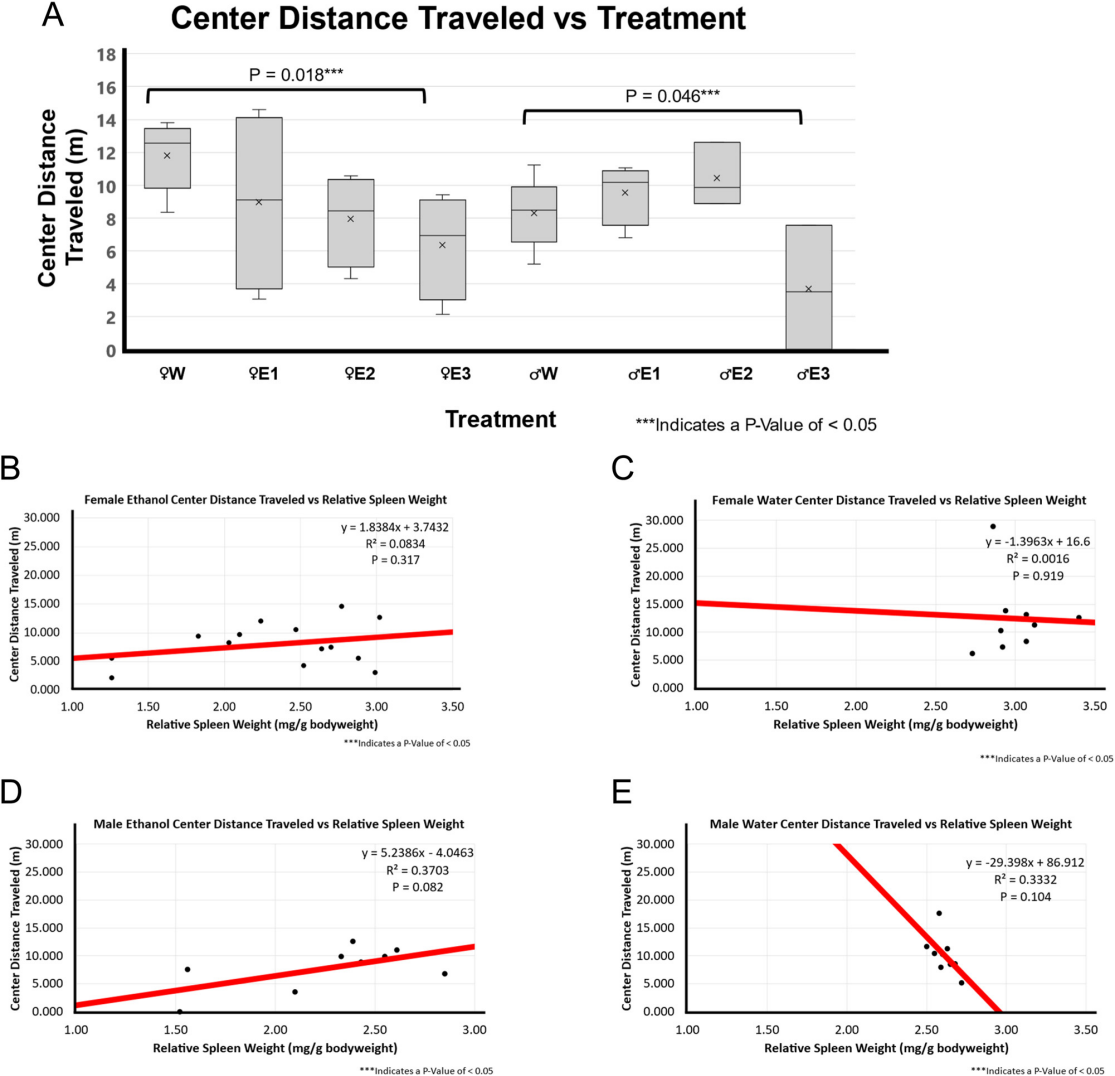
Distance traveled in the center zone and correlation with relative spleen weight following 3-day binge EtOH exposure. (A) The relative spleen weight between the treatment groups was compared using a *t*-test between water (n=5 for male and n=5 for female) and EtOH-treated rats for both males (n=10) and females (n=12). In females, animals with smallest spleen weight following EtOH exposure, E3 showed significantly less time in the center zone compared to female water-treated group (p=0.018). In males, smallest spleen group E3 group animals spent significantly less time in the center zone compared to male F344 rats treated with water (p=0.046). (B) A very weak positive correlation (r^2 of 0.0834; p=0.317; n=14) was found between relative spleen weight and distance traveled in the center zone in EtOH-treated female F344 rats. (C) A weak to no correlation (r^2 of 0.0016; p=0.919; n=9) between relative spleen weight and time mobile score was observed in water-treated female F344 rats. (D) Positive moderate correlation (r^2 of 0.3703; p=0.082; n=9) was found between smaller spleen weight and distance traveled in the center zone in EtOH-treated male F344 rats. (E) A negative correlation (r^2 of 0.3332; p-value of 0.104; n=9) was found between relative spleen weight and distance traveled in the center zone in water-treated male F344 rats.

In both female and male BE-treated groups, there was significantly less center distance traveled (female p-value of 0.018 and male p-value of 0.046) as shown in Figure 5A.

There was a weak neutral association between center distance traveled and RSW in the female BE-treated group (p-value of 0.317 and r^2 of 0.0834) and a very weak neutral association in the female water-treated group (p-value of 0.919 and r^2 of 0.0016) as shown in Figures 5B and 7C. There was little to no correlation between center distance traveled and RSW in females. Interestingly for males, there was a weak positive correlation in the BE-treated group (p-value of 0.082 and r^2 of 0.370) and a weak negative correlation in the water-treated group (p-value of 0.104 and r^2 of 0.333) as shown in Figure 5D and E. In BE-treated male rats, a smaller spleen weight was correlated with less center distance traveled while it was the opposite in water-treated rats with more center distance traveled.

### Modulation of anxiety and locomotor activity through the PFC given binge EtOH treatment

Of the 321 pathways that were identified to be significantly involved in the GSE49028 (p-value <0.05), 4 pathways were highlighted based on their role in anxiety (Serotonin Receptor Signaling) and locomotor deficit (Dopamine-DARPP32 Feedback in cAMP Signaling, Acetylcholine Receptor Signaling Pathway, and Glutamate Binding, Activation of AMPA Receptors and Synaptic Plasticity).

Figure 6 is the connectivity maps showing the regulation of the DEGs within the signaling pathways. The connectivity map depicted the regulation of the DEGs and the effects downstream of these DEGs within the signaling pathway. The up or down-regulation of the DEGs overlaid onto Serotonin Receptor Signaling revealed inhibition of serotonin receptor signaling associated with increased depressive-like behavior, anxiety, and apoptosis of neurons; and inhibition of neuronal excitation and survival of neurons as shown in Figure 6A. Figure 6B depicts the overlay of DEGs onto Dopamine-DARPP32 Feedback in cAMP Signaling with the inhibition of cell survival genes within the presynaptic neuron. The regulation of the DEGS within the Acetylcholine Receptor Signaling Pathway inhibited the survival of neurons and activated neuroinflammation and the anti-inflammatory response as shown in Figure 6C. In Figure 6D, the DEGs were overlayed onto the Glutamate Binding, Activation of AMPA Receptors, and Synaptic Plasticity pathway. Ca2+ and K+ transport was found to be activated suggesting dysregulation of the ion channels involved in depolarization.

**Figure 6A:**
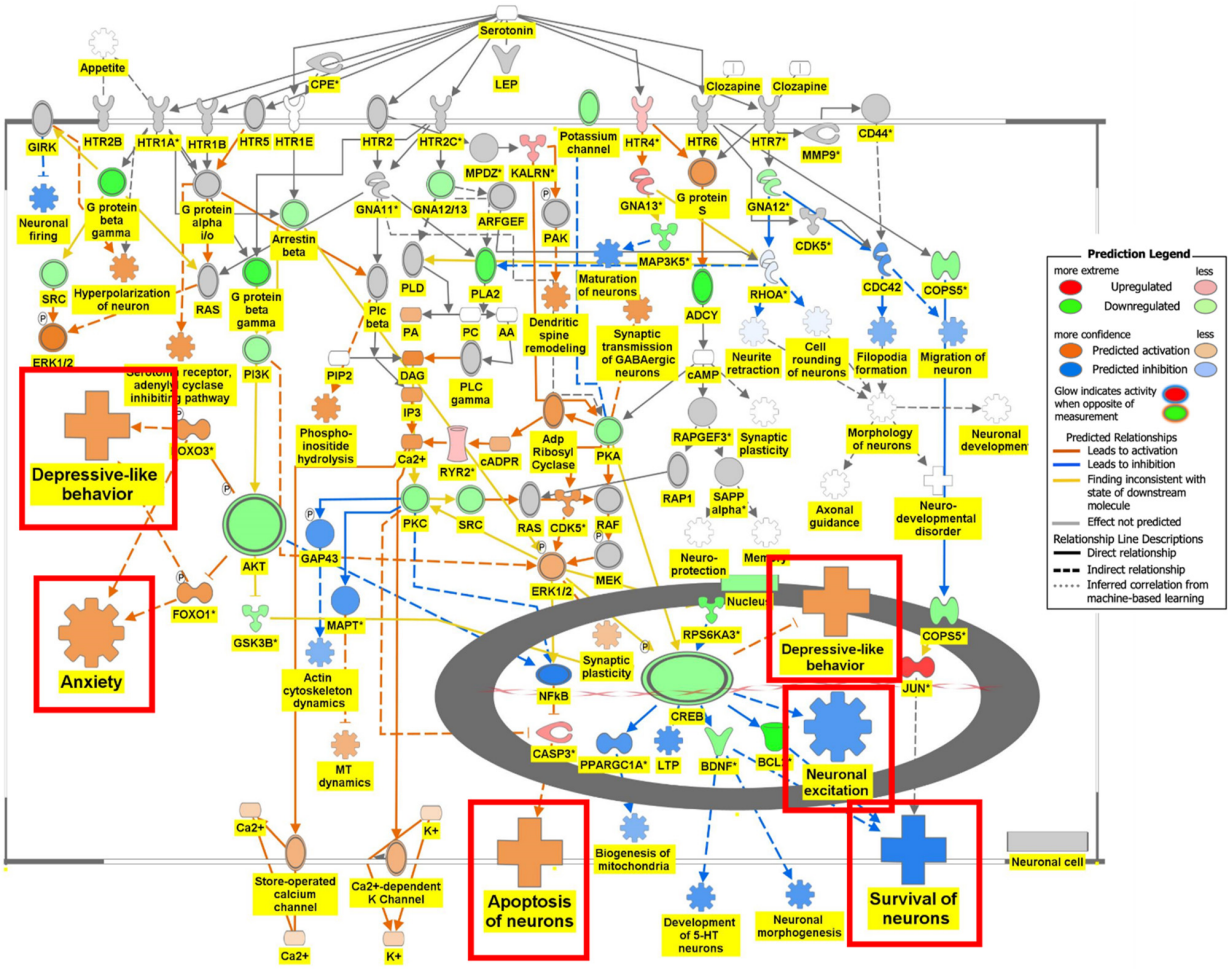
Regulation of DEGs from GSE49028 dataset of binge ethanol-treated mice on signaling pathways related to anxiety and locomotor activity. Regulation of serotonin signaling pathway by DEGs from GSE49028 through overlay of DEGs obtained from GSE49028 onto serotonin receptor signaling resulted in the activation of depressive-like behavior, anxiety, and apoptosis. However, neuronal excitation and survival of neurons were predicted to be inhibited (z-score activation=−0.198, −log(p-value)=2.67).

**Figure 6B:**
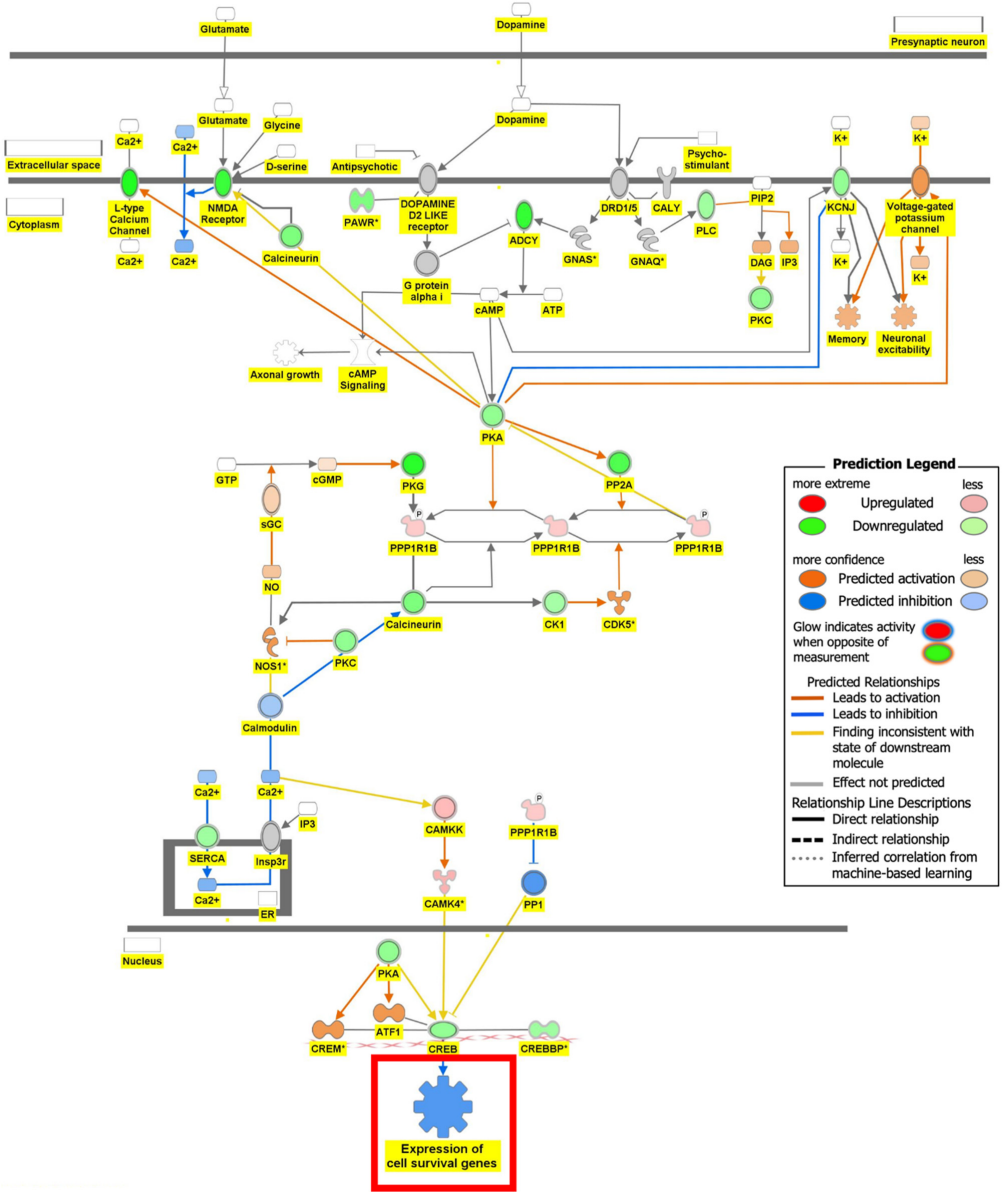
Regulation of DEGs from GSE49028 dataset of binge ethanol-treated mice on signaling pathways related to anxiety and locomotor activity. Regulation of dopamine-DARPP32 feedback in cAMP signaling through overlay of DEGs associated with acute alcohol exposure onto dopamine-DARPP32 feedback in cAMP signaling resulted inhibition of the signaling pathway and inhibited the function of expression of cell survival genes (z-score activation=−0.174, −log(p-value)=2.04).

**Figure 6C:**
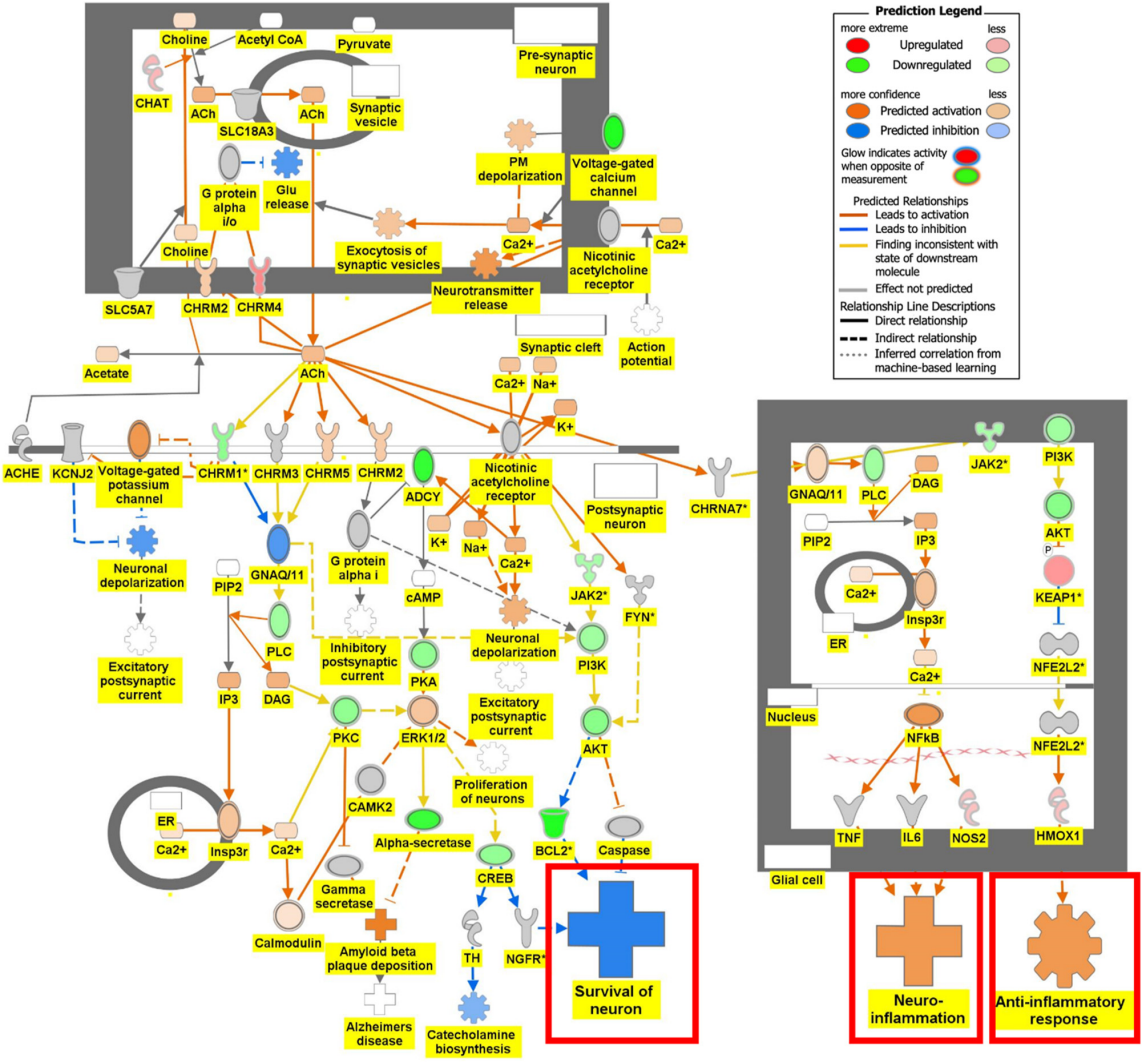
Regulation of DEGs from GSE49028 dataset of binge ethanol-treated mice on signaling pathways related to anxiety and locomotor activity. Regulation of acetylcholine receptor signaling pathway through overlay of DEGs onto acetylcholine receptor signaling pathway. Survival of neurons was predicted to be inhibited. Neuroinflammation and anti-inflammatory response were predicted to be activated (z-score activation=−0.302, −log(p-value)=1.72).

**Figure 6D:**
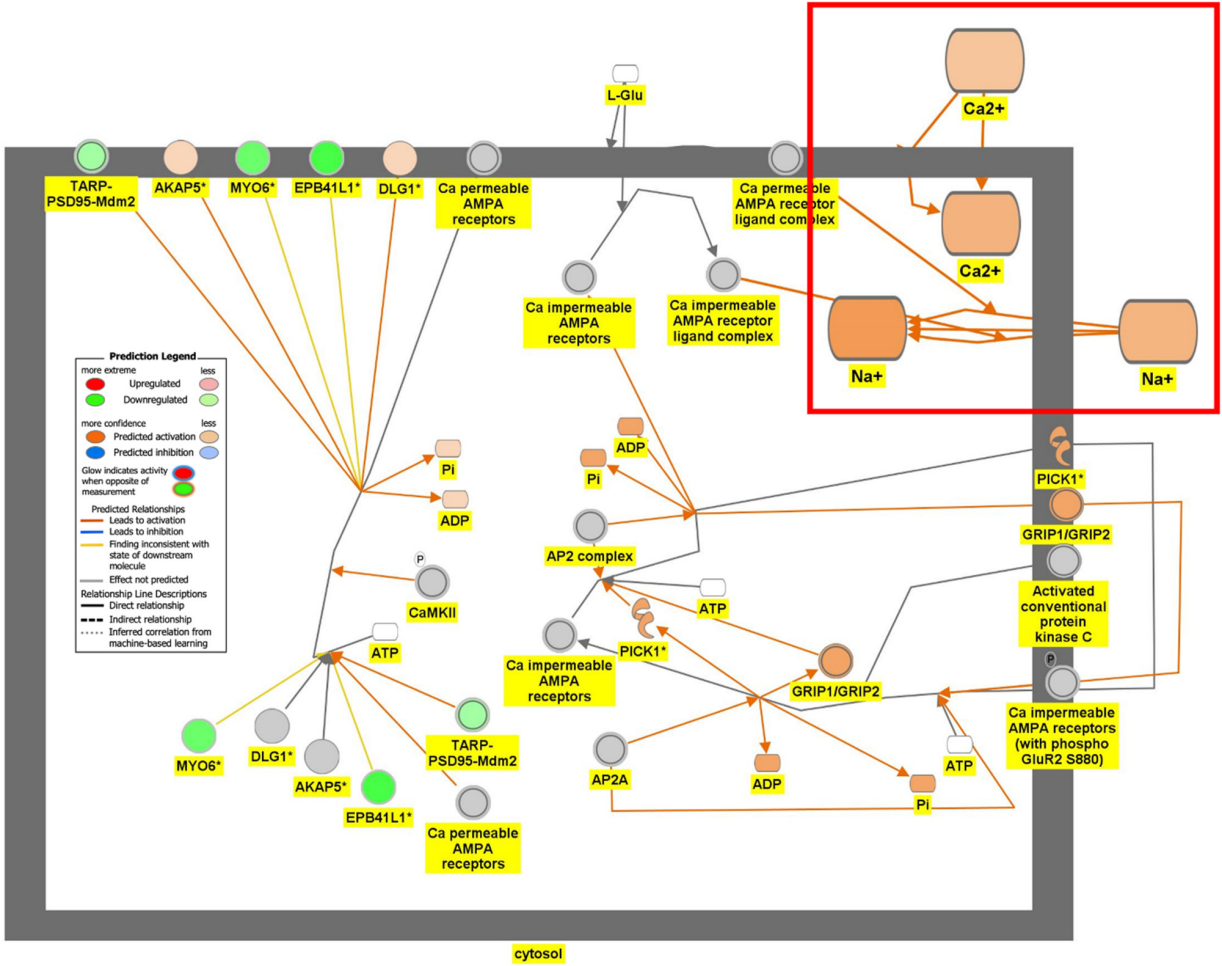
Regulation of DEGs from GSE49028 dataset of binge ethanol-treated mice on signaling pathways related to anxiety and locomotor activity. Regulation of glutamate binding, activation of AMPA receptors and synaptic plasticity through overlay of DEGs onto glutamate binding, activation of AMPA receptors and synaptic plasticity. Ca+ and K+ transport was predicted to be activated (z-score activation=1.265, −log(p-value)=1.41).

Functional analysis of the DEGs found the activation of anxiety, emotional behavior, cognitive impairment, and inhibition of exploratory behavior suggest increased anxiety. The inhibition of myelination of nerves and motor learning and the activation of motor dysfunction suggest locomotor deficits.

**Figure 7:**
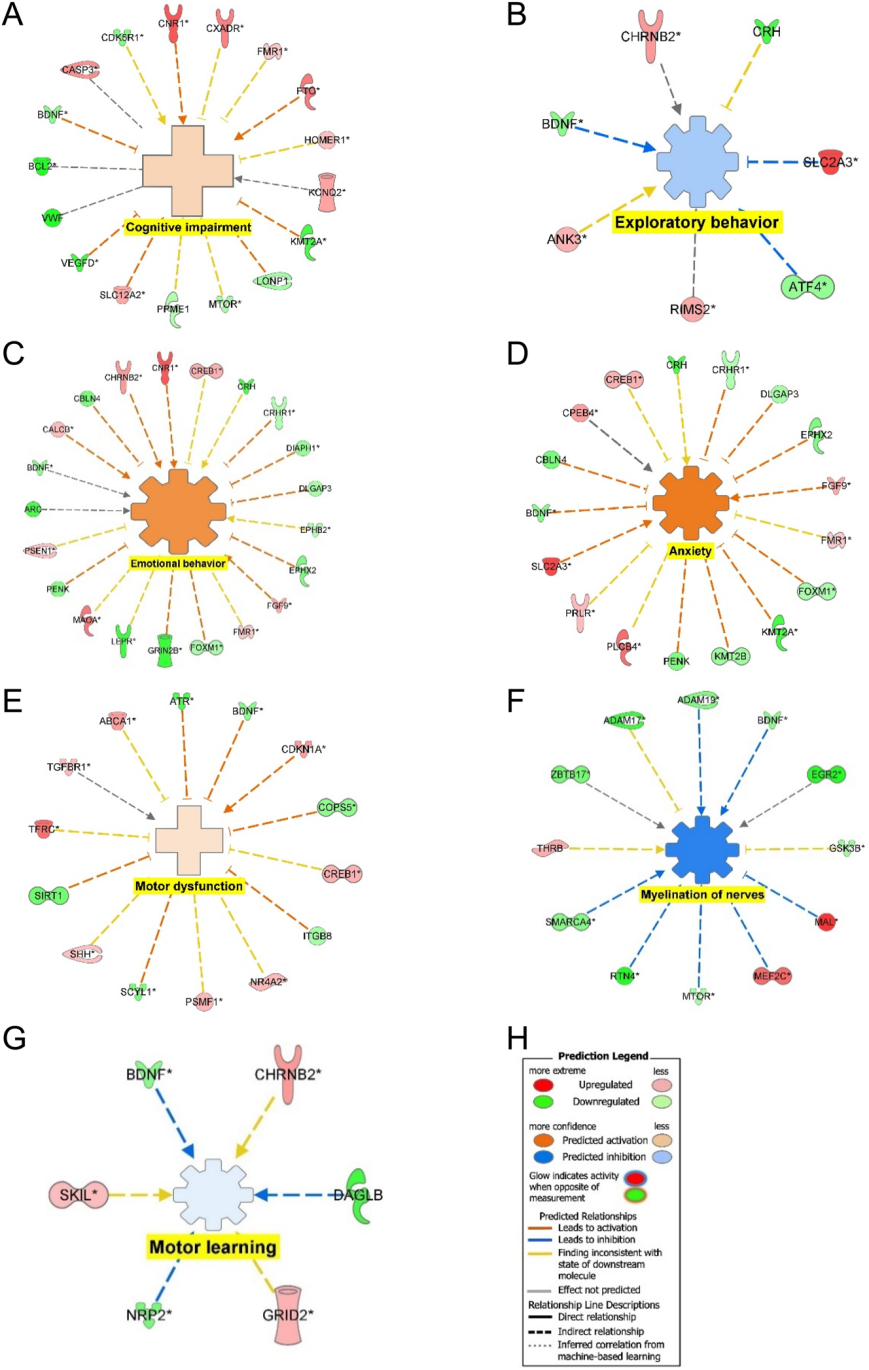
Regulation of DEGs from GSE49028 dataset of binge EtOH-treated mice on disease and biological functions related to anxiety and locomotor activity. (A) Regulation of DEGs leading to the activation of cognitive impairment (z-score activation=0.359, p-value=0.000463); (B) regulation of DEGs leading to inhibition of exploratory behavior (z-score activation=−0.447, p-value=0.00038); (C) regulation of DEGs leading to activation of emotional behavior (z-score activation=1.113, p-value=0.03680); (D) regulation of DEGs in leading to activation of anxiety (z-score activation=1.543, p-value=0.04120); (E) regulation of DEGs in leading to activation of motor dysfunction (z-score activation=0.249, p-value=0.001740); (F) regulation of DEGs in leading to inhibition of myelination of nerves (z-score activation=−1.353, p-value=0.003830); (G) regulation of DEGs in leading to inhibition of motor learning (z-score activation=−0.128, p-value=0.005550); and (H) legend.

Figure 7 shows the connectivity maps for the regulation of the DEGs within diseases or functions. CASP3, CNR1, CXADR, FMR1, FTO, HOMER1, KCNQ2, SLC12A2 were upregulated and BCL2, BDNF, CDKSR1, KMT2A, LONP1, MTOR, PPME1, VEGFD, and VWF were downregulated to mediate the activation of cognitive impairment (Figure 7A). The inhibition of exploratory behavior was mediated by ANK3, CHRNB2, RIMS2, and SLC2A3 were upregulated and ATF4, BDNF, and CRH were downregulated (Figure 7B). Figure 7C shows that CALCB, CHRNB2, CNR1, CREB1, FGF9, FMR1, MAOA, and PSEN1 were upregulated and ARC, BDNF, CBLN4, CRH, CRHNR1, DIAPH1, DLGAP3, EPHB2, EPHX2, FOXM1, GRIN2B, LEP3, and PENK were downregulated to mediate activation of emotional behavior. CPEB4, CREB1, FGF9, FMR1, PLCB4, PRLR, SLC2A3 were upregulated and BDNF, CBLN4, CRH, CRHR1, DLGAP3, EPHX2, FOXM1, KMT2B, PENK were downregulated to mediate the activation of anxiety (Figure 7D). The activation of motor dysfunction was mediated by the upregulation of ABCA1, CDKN1A, CREB1, NR4A2, PSMF1, SHH, TFRC, and TGFBR1 and downregulation of ATR, BDNF, COPSS, ITGB8, SCYL1, and SIRT1 (Figure 7E). Figure 7F shows that the upregulation of MAL, MEF2C, THRB were upregulated and ADAM17, ADAM19, BDNF, EDR2, GSK3B, MTOR, RTN4, SMARCA4, and ZBTB17 were downregulated to inhibit myelination of nerves. Motor learning was inhibited through the upregulation of CHRNB2, GRID2, and SKIL and the downregulation of BDNF, DAGLB, and NRP2 (Figure 7G). The regulation of DEGs in common that mediated the behavioral deficits from Figure 7A–G was the downregulation of BDNF.

## Discussion

In the present study, following 3-day BE treatment, F344 rats showed differential spleen atrophy correlated with behavioral deficits including motor deficits, fear and anxiety. The RSW and behavioral measurements were more affected in males than females. Bioinformatics tools enhance data analysis and hypothesis generation. We used IPA, a bioinformatic tool, to perform functional and pathway analysis of the DEGs in transcriptomic profile from the PFC of B6129SF2/J mice treated with BE or saline (GSE49028) to elucidate the effect of binge alcohol consumption on the brain. We identified several target pathways involved in mediating the behavioral deficits associated with binge drinking, aiding in the development of a potent cocktail of therapeutic drugs to target multiple signaling pathways in alcohol misuse pathologies.

The spleen is the largest peripheral lymphoid organ responsible for filtering blood, removing aged erythrocytes, and mediating immune responses [19]. We found differential BE-induced spleen atrophy in F344 rats. We observed sex-dependent effects of BE with males more sensitive than females. This is consistent with our previous finding that male rats were more sensitive to BE’s effects including body weight loss, spleen atrophy and abnormal immune cells [20]. We also reported that there are sex-dependent gene expression changes related to alcohol metabolism and epigenetic modifications between male and female rats given BE [7], 21], 22]. Sex dependence was also observed when we correlated the RSW with behavioral deficits, wherein a greater and stronger significant correlation in males than females (Table 1 **)**.

We found that the correlation between both total distance traveled and mean speed with RSW had a stronger positive correlation in males than in females (Table 1 **)**. BE-treated rats had less locomotor activity, suggesting motor deficits and impaired movement disorders [23]. Our correlation analysis suggests that BE treatment shows a more sensitive and stronger relationship between RSW and locomotor deficit in males than in females, where a smaller RSW change was correlated with greater behavioral deficits. Similar results were observed with mean speed (Figure 2A **)**. These findings suggest that EtOH induces a smaller spleen weight associated with locomotor deficits. There was a moderate positive correlation between RSW change and mean speed in EtOH-treated rats (Figure 2B–E). We found a stronger and more sensitive significant association between mean speed and RSW in EtOH-treated males than females, suggesting locomotor deficits are more prominent and stronger in EtOH-treated male rats with smaller RSWs.

To analyze the role of BE treatment on fear response, we measured freezing and mobility using the ANY-maze open-field assessment. A study using gelatin-based drinking in the dark model found that binge drinking led to acute movement impairments and anxiety-like behavior [24]. Time freezing, mean freezing score, and number of mobile episodes were significantly correlated with RSW in EtOH-treated males but not in EtOH-treated females, suggesting a sex-dependent difference in fear response shown in Table 1. The largest EtOH-induced RSW change was correlated with a stronger freezing response. A positive correlation was found between RSW and mean freezing score, with a prominent effect in male EtOH-treated group (Figures 3B, 3D, 4B, and D4D).

Thigmotaxis or wall hugging is associated with anxiety and less exploratory behavior. Rodents perceive large open areas as dangerous and tend to hug the walls as an anti-predatory mechanism [25]. The RSW in BE-treated males was significantly correlated with the number of center entries, the number of periphery entries, and the periphery distance traveled in a sex-dependent manner. In Figure 5A, both males and females with the greatest RSW change were significantly correlated with lower center distance traveled, suggesting that BE leads to greater anxiety and less exploratory behavior. These changes were prominent in BE-treated males than in females as shown in Figure 5B and D. Torcaso et al. found anxiety-like behavior using elevated plus maze in BE-treated peri-puberty male Wistar rats [26].

Sex-dependent differences may be due to the sexual dimorphism in the immune system and inflammatory responses [27]. Furthermore, we treated the rats when they were in a stage of adolescence with hormonal changes that can affect physical, cognitive, and emotional aspects. Female rats have faster alcohol metabolism than males which may be due to hormonal differences [28]. In addition, our previous study showed that there was higher expression of ALH1C and ALDH2 in adolescent female spleens compared to male spleens, suggesting faster alcohol metabolism which may contribute to the susceptibility of the male spleen to alcohol [7].

We found that a smaller spleen weight was correlated with greater anxiety and impaired locomotor activity, more profoundly in males than females, suggesting a sex-dependent effect of binge drinking. We analyzed the transcriptomic profile in PFC of binge EtOH-treated mice to provide insights into our behavioral analysis of the open-field assessment of binge EtOH-treated rats.

Binge drinking causes neuroinflammation and neuroimmune impairment mediated through the brain-spleen axis [8], 11]. Neuroimmune brain communication modulates stress through monocyte trafficking [13]. Our transcriptomic analysis identified several pathways of interest in BE’s effect on behavior. BE’s effect on anxiety may be mediated by the inhibition of Serotonin Receptor Signaling. Within the Serotonin Receptor Signaling pathway, the downstream effects of the DEGs in BE-treated mice were found to activate depressive-like behavior, anxiety, and apoptosis and inhibit neuronal excitation and survival of neurons through the upregulation of transcription factor CREB and downregulation of AKT as shown in Figure 6A.

Our pathway analysis provided us insight into the mechanisms underlying BE-associated behavioral deficits. BE’s impact on locomotor deficit may be mediated by dopamine-DARPP32 feedback in cAMP Signaling, Acetylcholine Receptor Signaling Pathway, and Glutamate Binding, Activation of AMPA Receptors and Synaptic Plasticity as shown in Figure 6B–D, respectively. The regulation of the DEGs within the Dopamine-DARPP32 Feedback in cAMP signaling and Acetylcholine Receptor Signaling pathway suggested that BE promoted presynaptic and post-synaptic neuron death, respectively. Within the Acetylcholine Receptor Signaling pathway, BE led to the activation of neuroinflammation and anti-inflammatory response by glial cells as shown in Figure 6C. Dysregulation of Ca2+ and K+ transport of glutamate signaling suggests impaired motor coordination and motor deficits [29]. These pathways will help develop therapeutic agents that target multiple signaling pathways.

Functional analysis provides insight into the regulation of DEGs on biological diseases and functions associated with BE within the PFC. Cognitive impairment, emotional behavior, and anxiety were activated within the PFC of BE-treated mice, supporting our finding of increased anxiety and fear response in BE-treated rats. Exploratory behavior was inhibited supporting thigmotaxis behavior observed in BE-treated rats as shown in Figure 7. Figure 7 also found the motor deficits through the activation of motor dysfunction and inhibition of myelination of nerves and motor learning, supporting our behavioral analysis of the increased freezing response and motor deficits associated with BE treatment. Figure 7A–G shows downregulation of BDNF as a common DEG among the various behavior deficit connectivity maps of BE-regulated DEGs.

## Conclusions

We observed that binge drinking induced differential spleen weight changes correlating with motor deficits, anxiety and fear in a sex-dependent manner with males more than females. Bioinformatic analysis identified key pathways (Serotonin Receptor Signaling, Dopamine-DARPP32 Feedback in cAMP Signaling, Acetylcholine Receptor Signaling Pathway, and Glutamate Binding, Activation of AMPA Receptors and Synaptic Plasticity) related to increased anxiety and impaired locomotion in BE-treated young adult mice. These findings revealed potential therapeutic targets for alcohol-induced spleen atrophy and behavioral deficits. By combining data-driven discovery with hypothesis-driven investigation, we have integrated these two studies to uncover the biological mechanisms linking BE-induced spleen atrophy to behavioral impairments, particularly through genetic alterations in the PFC. Our findings pave the way for developing potent treatments to address behavioral impairments in binge drinking individuals.

## Supplementary Material

Supplementary Material Details
