# Supplementary material for: Sex dependent correlation of spleen atrophy and behavior deficits induced by binge treatment with ethanol in rodent models
Source: NeuroImmune Pharm Ther. 2024 Dec 11;4(1):59–75. doi: 10.1515/nipt-2024-0016 (PMC12041846; doi:10.1515/nipt-2024-0016)
Supplement: Supplementary file 1 — Supplementary Material Details [file j_nipt-2024-0016_suppl_001.docx]

**Abbreviation Table**

| **Abbreviation** | **Full Term** |
| --- | --- |
| ABCA1 | ATP binding cassette subfamily A member 1 |
| ADAM17 | ADAM metallopeptidase domain 17 |
| ADAM19 | ADAM metallopeptidase domain 19 |
| ANK3 | ankyrin 3 |
| ARC | activity regulated cytoskeleton associated protein |
| ATF4 | activating transcription factor 4 |
| ATR | ATR serine/threonine kinase |
| BAC | Blood alcohol concentration |
| BCL2 | BCL2 apoptosis regulator |
| BDNF | brain derived neurotrophic factor |
| BE | Binge treatment with EtOH |
| CALCB | calcitonin related polypeptide beta |
| CASP3 | caspase 3 |
| CBLN4 | cerebellin 4 precursor |
| CDK5R1 | cyclin dependent kinase 5 regulatory subunit 1 |
| CDKN1A | cyclin dependent kinase inhibitor 1A |
| CHRNB2 | cholinergic receptor nicotinic beta 2 subunit |
| CNR1 | cannabinoid receptor 1 |
| CNS | Central nervous system |
| COPS5 | COP9 signalosome subunit 5 |
| CPEB4 | cytoplasmic polyadenylation element binding protein 4 |
| CREB1 | cAMP responsive element binding protein 1 |
| CRH | corticotropin releasing hormone |
| CRHR1 | corticotropin releasing hormone receptor 1 |
| CXADR | CXADR Ig-like cell adhesion molecule |
| DAGLB | diacylglycerol lipase beta |
| DEGs | Differentially Expressed Genes |
| DIAPH1 | diaphanous related formin 1 |
| DLGAP3 | DLG associated protein 3 |
| EGR2 | early growth response 2 |
| EPHB2 | EPH receptor B2 |
| EPHX2 | epoxide hydrolase 2 |
| EtOH | Ethanol |
| FemaleE1 | Female group with low relative spleen weight change |
| FemaleE2 | Female group with moderate relative spleen weight change |
| FemaleE3 | Female group with high relative spleen weight change |
| FGF9 | fibroblast growth factor 9 |
| FMR1 | fragile X messenger ribonucleoprotein 1 |
| FOXM1 | forkhead box M1 |
| FTO | FTO alpha-ketoglutarate dependent dioxygenase |
| GEO | Gene Expression Omnibus |
| GRID2 | glutamate ionotropic receptor delta type subunit 2 |
| GRIN2B | glutamate ionotropic receptor NMDA type subunit 2B |
| GSK3B | glycogen synthase kinase 3 beta |
| HOMER1 | homer scaffold protein 1 |
| HPA | Hypothalamic-pituitary-adrenal |
| IACUC | Institutional Animal Care and Use Committee |
| IPA | Ingenuity Pathway Analysis |
| ITGB8 | integrin subunit beta 8 |
| KCNQ2 | potassium voltage-gated channel subfamily Q member 2 |
| KMT2A | lysine methyltransferase 2A |
| KMT2B | lysine methyltransferase 2B |
| LEPR | leptin receptor |
| LONP1 | lon peptidase 1, mitochondrial |
| MAL | mal, T cell differentiation protein |
| MaleE1 | Male group with low relative spleen weight change |
| MaleE2 | Male group with moderate relative spleen weight change |
| MaleE3 | Male group with high relative spleen weight change |
| MAOA | monoamine oxidase A |
| MEF2C | myocyte enhancer factor 2C |
| MTOR | mechanistic target of rapamycin kinase |
| NIAAA | National Institute on Alcohol Abuse and Alcoholism |
| NR4A2 | nuclear receptor subfamily 4 group A member 2 |
| NRP2 | neuropilin 2 |
| PENK | proenkephalin |
| PFC | Prefrontal cortex |
| PLCB4 | phospholipase C beta 4 |
| PND | Postnatal day |
| PPME1 | protein phosphatase methylesterase 1 |
| PRLR | prolactin receptor |
| PSEN1 | presenilin 1 |
| PSMF1 | proteasome inhibitor subunit 1 |
| RIMS2 | regulating synaptic membrane exocytosis 2 |
| RSW | Relative spleen weight |
| RTN4 | reticulon 4 |
| SCYL1 | SCY1 like pseudokinase 1 |
| SHH | sonic hedgehog signaling molecule |
| SIRT1 | sirtuin 1 |
| SKIL | SKI like proto-oncogene |
| SLC12A2 | solute carrier family 12 member 2 |
| SLC2A3 | solute carrier family 2 member 3 |
| SMARCA4 | SWI/SNF related, matrix associated, actin dependent regulator of chromatin, subfamily a, member 4 |
| TFRC | transferrin receptor |
| TGFBR1 | transforming growth factor beta receptor 1 |
| THRB | thyroid hormone receptor beta |
| VEGFD | vascular endothelial growth factor D |
| VWF | von Willebrand factor |
| ZBTB17 | zinc finger and BTB domain containing 17 |
